# The effect of material assignment in nasal cavity on dose calculation for nasopharyngeal carcinoma (NPC) using Acuros XB

**DOI:** 10.1002/acm2.13698

**Published:** 2022-06-14

**Authors:** Michael L. M. Cheung, Vivian U. Y. Chow, Monica W. K. Kan, Anthony T. C. Chan

**Affiliations:** ^1^ Department of Clinical Oncology Prince of Wales Hospital Hong Kong SAR China; ^2^ Department of Clinical Oncology The Chinese University of Hong Kong Hong Kong SAR China

**Keywords:** Acuros XB algorithm, dose calculation, inhomogeneity, material assignment, nasopharyngeal carcinoma

## Abstract

**Purpose:**

To evaluate the effect of material assignment in nasal cavity on dose calculation for the volumetric modulated arc therapy (VMAT) of nasopharyngeal carcinoma (NPC) using Acuros XB (AXB) algorithm.

**Methods:**

The VMAT plans of 30 patients with NPC were calculated using AXB with material auto‐assignment of nasal cavity to lung and reassignment to air respectively. The doses to the planning target volumes (PTVs) overlapping with nasal cavity with material auto‐assignment of lung (AXB_Lung) were compared to the values obtained when nasal cavity was reassigned to air (AXB_Air) under the dose‐to‐medium (*D*_m_) reporting mode of AXB.

**Results:**

For dose calculated under AXB_Lung, the *D*_98%_, *D*_2%_, and *D*_mean_ of the PTV_69.96__Air Cavity (PTV of prescription dose 69.96 Gy overlapping with nasal cavity) were on average 16.1%, 1.6%, and 8.6% larger than that calculated under AXB_Air, respectively. Up to 19.5% difference in *D*_98%_, 3% difference in *D*_2%_, and 11.2% difference in *D*_mean_ were observed in the worst cases for PTV_69.96_. Similar trend was observed for the PTV_5940__Air Cavity, in which the *D*_98%_, *D*_2%_, and *D*_mean_ calculated under AXB_Lung were on average 14.7%, 2.5%, and 10.2% larger than that calculated under AXB_Air, respectively. In the worst cases, the difference observed in *D*_98%_, *D*_2%_, and *D*_mean_ could be up to 17.7%, 4.5%, and 12.7%, respectively.

**Conclusions:**

Significant dose difference calculated by AXB between the material assignment of lung and air in nasal cavity for NPC cases might imply the possibility of underdosage to the PTVs that overlap with inhomogeneity. Therefore, attention should be put to ensure that accurate material assignment for dose calculation under AXB such that optimal dosage was given for tumor control.

## 1 INTRODUCTION

Volumetric modulated arc therapy (VMAT) is one of the most common treatment modalities for nasopharyngeal carcinoma (NPC) due to its ability to produce highly conformal dose distributions to the target while minimizing doses to organs‐at‐risk.^1^ As the nasopharyngeal region is surrounded by heterogeneous medium such as air cavities, the accuracy of dose calculation for these NPC cases is significantly affected by the ability of algorithms to account for electron transport in air–tissue interface.^2^ Anisotropic analytical algorithm (AAA) and collapsed cone convolution (CCC) are widely used convolution/superposition algorithms implemented in commercial treatment planning system. They apply the simplified density scaling of the Monte Carlo (MC)‐derived dose kernels to account for the presence of inhomogeneities so that the secondary electron transport is only modeled macroscopically. Previous studies found that AAA and CCC significantly overpredict the dose near air–tissue interfaces.

A more advanced dose calculation algorithm known as Acuros XB (AXB) that can achieve comparable accuracy with MC has been implemented in Eclipse treatment planning system (Varian Medical Systems, Palo Alto, CA, USA). It is a deterministic solver of linear Boltzmann transport equation (LBTE) describing the macroscopic behavior of ionizing particles as they travel through and interact with matter.^3^ By solving the LBTE, the electron  fluence is obtained and the dose is generated by using macroscopic electron energy deposition cross sections and the density of materials. In AXB, two dose reporting options are provided, including dose‐to‐water (*D*_w_) and dose‐to‐medium (*D*_m_). Both options are the same for AXB transport calculation, in which the electron fluence is calculated based on the material properties of crossing media. The main difference arises from post‐processing step, in which the electron fluence is multiplied by a water‐based flux‐to‐dose response function and medium‐based flux‐to‐dose response function for *D*_w_ and *D*_m_, respectively.^2^, ^4^, ^5^ Therefore, the material composition of voxels in computed tomography (CT) images is required for dose calculation. The material is assigned automatically by the conversion of Hounsfield units to mass density using CT calibration curve, followed by looking up the material in the Varian system database.^6^ For those voxels with density larger than 3.0 g/cm^3^, manual material assignment is required in Eclipse to prevent the inaccurate assignment to high‐density materials such as metallic prosthesis.^6^, ^7^

Several publications have reported that AXB provides an accurate dose calculation in heterogeneous environment.^5^, ^7^, ^8^, ^9^ However, no study has been conducted to evaluate the effect of material assignment on dose calculation in the presence of air cavity in nasopharyngeal region using real clinical cases. Therefore, the aim of this study was to assess the dosimetric impact of material assignment to nasal cavity under AXB using real clinical NPC cases.

## 2 METHODS

### 2.1 Contouring and prescription of target volumes

**FIGURE 1:**
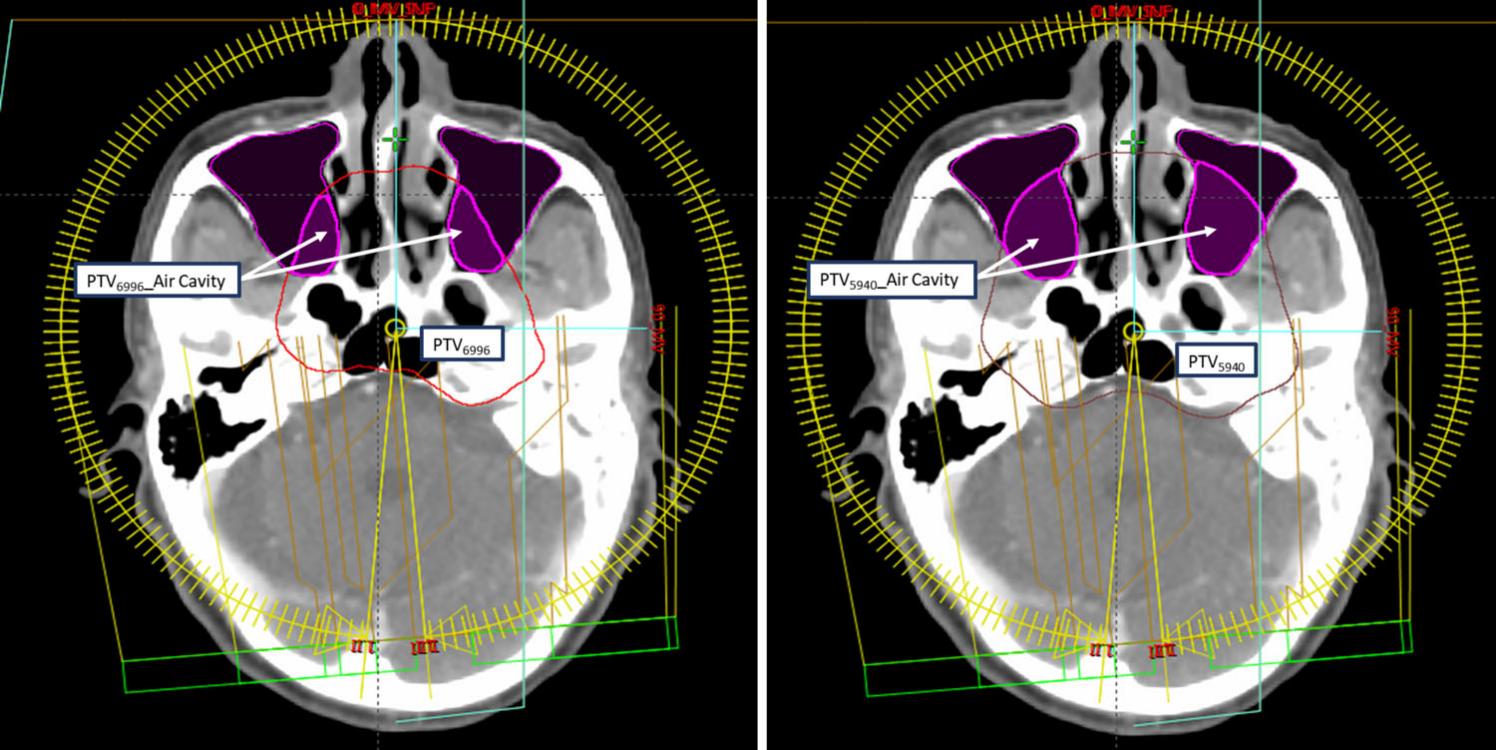
Contouring for the dosimetric evaluation of material assignment in nasal cavity. (Left) PTV_6996_ was in red contour, and its overlapping part with nasal cavity was PTV_6996__Air Cavity. (Right) The brown contour was PTV_5940_, and its overlapping part with nasal cavity was PTV_5940__Air Cavity.

A total of 30 NPC VMAT patients with treatment site overlapping nasal cavity were selected for retrospective analysis. All plans were generated using a 6‐MV beam and modulated with a 120 multi‐leaf collimator (MLC) from TrueBeam linear accelerator (Varian Medical Systems, Palo Alto, CA, USA). The target volume of each patient was defined by the oncologist using 3‐mm‐thick axial CT images. The planning target volume (PTV) included the abnormal soft tissue mass with an addition of 5‐mm margin to account for organ movement and patient setup uncertainty. The mean PTV was 313.2 cm^3^ (ranging from 104.4 to 804.1 cm^3^) and 921.9 cm^3^ (ranging from 504.7 to 1590 cm^3^) for prescription dose 69.96 Gy (PTV_6996_) and 59.40 Gy (PTV_5940_), respectively. To evaluate the dosimetric difference of material assignment to nasal cavity, three additional structures were contoured, including the whole nasal cavity (Air Cavity), PTV of prescription dose 69.96 Gy overlapping with nasal cavity (PTV_6996__Air Cavity), and PTV of prescription dose 59.40 Gy overlapping nasal cavity (PTV_5940__Air Cavity), as shown in Figure 1.

### 2.2 Treatment planning

The treatment plans were generated using treatment planning system Eclipse version 13.6 (Varian Medical Systems, Palo Alto, CA, USA). At least 95% of the PTVs received the prescription dose with the optimization criteria followed NRG‐HN001 protocol.^10^ Each treatment plan was then calculated using AXB version 13.6.23 (Varian Medical Systems, Palo Alto, CA, USA) with automatic material assignment of nasal cavity to low‐density lung in Eclipse. After that, the Air Cavity was manually assigned to the material of air, and the plans were recalculated with the identical treatment parameters such as monitor unit and MLC setting using AXB. In present study, AXB using *D*_m_ option with 2.5 mm dose grid resolution was used.

### 2.3 Dosimetric evaluation

The doses PTV_6996__Air Cavity and PTV_5940__Air Cavity with material auto‐assignment of lung (AXB_Lung) were compared to the values obtained when nasal cavity was reassigned to air (AXB_Air). The *D*_mean_, *D*_2%_ (minimum dose received by 2% of PTV volume), and *D*_98%_ (minimum dose received by 98% of PTV volume) of the Air Cavity, PTV_6996__Air Cavity, and PTV_5940__Air Cavity were compared. Statistical analyses were performed using SPSS Statistics version 17.0 (SPSS, Inc., Chicago, IL, USA) in this study. Paired *t*‐test was conducted to investigate if there was significant difference in the dose calculation. The statistical test was two‐sided, and *p*‐value <0.05 was considered statistically significant.

## 3 RESULTS

The comparison between doses received by Air Cavity, PTV_6996__Air Cavity, and PTV_5940__Air Cavity under AXB_Air and AXB_Lung was shown in Table 1. For dose calculated under AXB_Lung, the *D*_98%_, *D*_2%_, and *D*_mean_ of the PTV_6996__Air Cavity were on average 16.1%, 1.6%, and 8.6% larger than that calculated under AXB_Air, respectively. Up to 19.5% difference in *D*_98%_, 3% difference in *D*_2%_, and 11.2% difference in *D*_mean_ were observed in the worst cases for PTV_6996__Air Cavity.

**TABLE 1:** Comparison of dose calculated by Acuros XB (AXB) under AXB_Air and AXB_Lung

		Dose difference (%)			
Structure	Parameters (Gy)	Mean ± SD	Min	Max	*p*‐Value*
Air Cavity	*D*_mean_	8.7 ± 1.3	6.0	12.8	<0.001**
	*D*_2%_	3.5 ± 1.6	7.1	0.5	<0.001**
	*D*_98%_	2.4 ± 1.4	5.5	0.5	<0.001**
PTV_6996__Air Cavity	*D*_mean_	8.6 ± 1.8	2.2	11.2	<0.001**
	*D*_2%_	1.6 ± 0.7	0.2	3.0	<0.001**
	*D*_98%_	16.1 ± 2.7	4.8	19.5	<0.001**
PTV_5940__Air Cavity	*D*_mean_	10.2 ± 1.4	6.9	12.7	< 0.001**
	*D*_2%_	2.5 ± 1.1	0.3	4.5	< 0.001**
	*D*_98%_	14.7 ± 2.4	4.0	17.7	< 0.001**

* The *p*‐value was calculated using paired *t*‐test.

** The *p*‐value has statistical significance of difference (*p* < 0.05).

Similar trend was observed for the PTV_5940__Air Cavity, in which the *D*_98%_, *D*_2%_, and *D*_mean_ calculated under AXB_Lung were on average 14.7%, 2.5%, and 10.2% larger than that calculated under AXB_Air, respectively. In the worst cases, the difference observed in D_98%_, D_2%_, and *D*_mean_ could be up to 17.7%, 4.5%, and 12.7%, respectively. Paired *t*‐test showed that all the dose differences with material assignment of lung and air were statistically significant (*p* < 0.05).

## 4 DISCUSSION

The dosimetric effect owning to inhomogeneities such as air cavity is one of the major concerns for NPC cases, and AXB was proved to be able to achieve comparable accuracy as the MC method in a heterogeneous medium. Although AXB is capable of providing accurate dose calculation, its accuracy depends on the preciseness of material assignment in AXB.^11^, ^12^ The material composition of each voxel in CT image was assigned automatically from the conversion of mass density from CT value based on the CT calibration curve, followed by looking up the material from material data table in AXB. Fogliata et al. showed that the dose computed inside the air material layer by AXB version 11 presents much better agreement with MC, due to the inclusion of air material assignment that was considered as lung in version 10.^7^, ^8^, ^11^ In AXB version 13.6.23, the automatic assignment of materials includes human material such as “Air,” “Lung,” “Adipose Tissue,” “Muscle, Skeletal,” “Cartilage,” and “Bone.” The density ranges for each material are slightly overlapping as shown in Table 2.^3^

**TABLE 2:** Look‐up table in Acuros XB (AXB) version 13.6.23 for automatic material assignment

Materials	Density range (g/cm^3^)
Air	0.000–0.020
Lung	0.011–0.624
Adipose tissue	0.554–1.001
Muscle, skeletal	0.969–1.093
Cartilage	1.056–1.600
Bone	1.100–3.000

In present study, the nasal cavity region was contoured and auto‐assigned with the material of “Lung” for dose calculation in Eclipse. As the nasal cavity is an air‐filled structure, the nasal cavity region was reassigned to “Air” and recalculated using AXB. Our study showed that the calculated *D*_mean_ and *D*_98%_ of PTV_6996__Air Cavity and PTV_5940__Air Cavity under AXB_Lung were much higher than that under AXB_Air. It implied that the dose reported for PTV_6996__Air Cavity under AXB_Lung might be overestimated for an air‐filled nasal cavity, leading to the possibility of underdosage or insufficient coverage to PTV_6996_. According to AAPM Task Group Report 105, 5% change in dose can result in 10%–20% change in tumor control probability or up to 20%–30% change in normal tissue complication probability if the prescribed dose is within the steepest region of dose–effect curves.^9^, ^13^ Therefore, attention should be put to ensure the accuracy of material assignment for dose calculation in AXB such that optimal dosage was given for tumor control.

Apart from the inherent inhomogeneity (i.e., air cavity) in NP region, imaging artifacts in planning CT and implants inside the patients with density less than 3.0 g/cm^3^ might also cause the misrepresentation of density information and hence leading to misassignment of materials for AXB dose calculation.^14^ Review of material assignment is therefore recommended when using AXB for dose calculation under *D*_m_.

## 5 CONCLUSION

Although the dose calculation of AXB was superior to other superposition/convolution methods in heterogeneous media, its accuracy is greatly affected by the material assignment. Therefore, a review of automatic material assignment is therefore recommended for accurate dose calculation such that the PTVs are adequately covered with optimal dose for tumor control.

## CONFLICT OF INTEREST

The authors declare that there is no conflict of interest that could be perceived as prejudicing the impartiality of the research reported.

## AUTHOR CONTRIBUTION

Michael L. M. Cheung was responsible for idea conceptualization, research design, literature review, data collection, data analysis, and manuscript writing. Vivian U. Y. Chow was responsible for research design, data collection, data analysis, and manuscript editing. Monica W. K. Kan and Anthony T. C. Chan were responsible for research guidance and supervision.
